# Spatial distribution and ecological niches of non-breeding planktivorous petrels

**DOI:** 10.1038/srep12164

**Published:** 2015-07-13

**Authors:** Joan Navarro, Laura Cardador, Ruth Brown, Richard A. Phillips

**Affiliations:** 1 Department of Conservation Biology, Estación Biológica de Doñana CSIC, Sevilla 41092, Spain; 2 British Antarctic Survey, Natural Environment Research Council, Cambridge CB3 0ET, UK

## Abstract

According to niche theory, mechanisms exist that allow co-existence of organisms that would otherwise compete for the same prey and other resources. How seabirds cope with potential competition during the non-breeding period is poorly documented, particularly for small species. Here we investigate for the first time the potential role of spatial, environmental (habitat) and trophic (isotopic) segregation as niche-partitioning mechanisms during the non-breeding season for four species of highly abundant, zooplanktivorous seabird that breed sympatrically in the Southern Ocean. Spatial segregation was found to be the main partitioning mechanism; even for the two sibling species of diving petrel, which spent the non-breeding period in overlapping areas, there was evidence from distribution and stable isotope ratios for differences in habitat use and diving depth.

An enduring constraint for many marine predators forced to return to land to breed is how to locate enough resources for maintenance and reproduction within an economical commuting distance from the colony. Particularly for colonial breeders, competition for trophic resources is intense, and many studies of nesting seabirds have provided evidence of ecological segregation by a variety of mechanisms^1^,^2^. By comparison, the means by which seabirds avoid potential competition during the non-breeding period are poorly documented, and the limited research to-date has focused on large species^3^,^4^,^5^.

In terms of numerical abundance and the biomass of prey required to sustain them, many seabird communities are dominated by small species. In the sub-Antarctic, for example, small petrels (<250 g) consume ~1 million tonnes of crustaceans per year^6^. Their dependence on broadly the same type of prey is considered the main reason for the interspecific differences in distribution and activity patterns observed in this feeding guild during the breeding season^2^,^7^. Whether the same mechanisms reduce competition during the non-breeding season is less clear, largely because of the practical difficulties involved in investigating habitat use and foraging behaviour of multiple species from the same community when no longer under central-place constraints. Hence, much of the available evidence for differences in feeding strategies in small seabirds during the non-breeding season is based on proxies, including stable isotope ratios in tissues collected when birds eventually return to colonies^7^,^8^.

Here we investigated for the first time the potential ecological isolating mechanisms that might operate during the non-breeding period of four small (120–200 g), very abundant zooplanktivorous seabirds - blue petrel (*Halobaena caerulea*), Antarctic prion (*Pachyptila desolata*), common diving petrel (*Pelecanoides urinatrix*) and South Georgian diving petrel (*P. georgicus*) - which breed in sympatry on islands in the Southern Ocean. We examined spatial movements, environmental (habitat) and trophic niches by analysing tissue stable isotope ratios, and integrating distribution of birds tracked using miniaturized geolocators with remotely-sensed oceanographic variables.

## Results

### Spatial distribution

**Figure 1:**
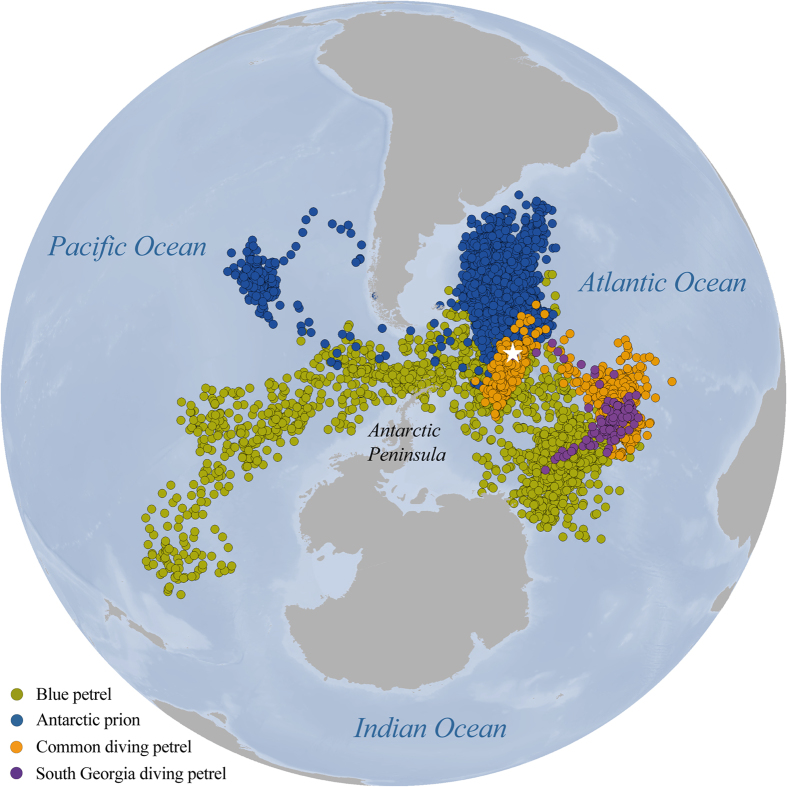
Locations of blue petrels, Antarctic prions, South Georgia diving petrels and common diving petrels from South Georgia (white star) tracked using geolocators during the non-breeding season in 2011 (the map is made by ArcGIS 9.3.1 software, http://www.arcgis.com/features).

**Figure 2:**
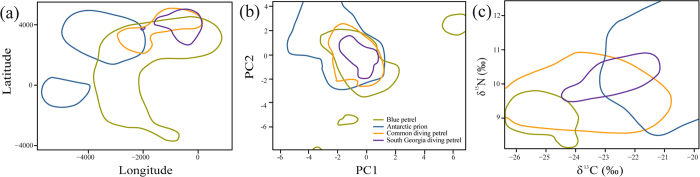
(**a**) Spatial (tracking locations), (**b**) habitat niche (PCA analysis) and (**c**) isotopic niche overlap between blue petrels, Antarctic prions, South Georgia diving petrels and common diving petrels from South Georgia. Spatial and habitat niche were calculated from individuals tracked using geolocators during the non-breeding season in 2011.

Spatial overlap was very low and non-significant for all species, except the two diving petrels (Table 1, Figs 1 and 2a). Blue petrels occupied a broad swathe of Antarctic waters in the Atlantic and Pacific oceans, with two areas of high density in the south and southeast of South Georgia (Fig. 1). Antarctic prions were also distributed in Atlantic and, to a lesser extent, Pacific waters, but at lower latitudes than blue petrels (Fig. 1). South Georgia and common diving petrels remained in the Atlantic, either close to South Georgia or in an area to the east-northeast (Fig. 1).

### Habitat niche

The Principal Component Analysis generated 8 different principal components (PCs), with the two first PCs, PC1 and PC2, explaining 39.3% and 26.5% of the total variance, respectively (see Table S1 and Figure S1 in the electronic supplementary material). PC1 represents a gradient from warm to cold waters (based on SST) and shallow depths, and PC2, a gradient from low salinity and low chl-a to warmer waters far from the ice shelf (see Table S1 and Figure S1 in the electronic supplementary material). Habitat niche overlap was higher than spatial overlap and was statistically significant when habitat availability was taken into account (with the exception of the two diving petrels, Fig. 2b; Table 1).

### Isotopic space

δ^13^C and δ^15^N differed among species (F_3,45_ = 12.99 p < 0.0001, and F_3,45_ = 8.96, p < 0.0001, respectively). Posthoc tests indicated that values for δ^15^N and δ^13^C were significantly highest in Antarctic prions, followed by South Georgia diving petrels and common diving petrels (which differed from each other in δ^15^N but not δ^13^C), and significantly lowest in blue petrels (Fig. 2c). Isotopic overlap varied among species, but was low overall, and non-significant (Fig. 2c; Table 1).

## Discussion

Our study revealed divergent movement and foraging strategies adopted by these four small sympatric seabird species when they disperse away from the colony during the non-breeding season. Segregation in geographic space was evident for five of the six paired comparisons between the four species (i.e. all but that between the two diving petrels). There was more overlap in habitat use (environmental niche), at least at the spatial resolution of GLS data, from which we conclude that spatial segregation is the more important mechanism for reducing competition among these species^1^,^2^,^9^,^10^.

Non-breeding blue petrels and Antarctic prions were clearly segregated in geographic space. Whereas blue petrels used Antarctic waters in the Atlantic and Pacific oceans, Antarctic prions were distributed largely in subantarctic waters east of the Patagonian Shelf (the latter consistent with previous tracking results^11^). The differences in isotopic composition of feathers provided further evidence for spatial segregation. Given the variation between water masses in baseline isotope ratios in the South Atlantic, the high δ^13^C of Antarctic prions corresponds well with their use of warmer waters to as far north as the subtropical zone, and the low δ^13^C of blue petrels to cold, Antarctic waters^8^,^12^,^13^. There may also be differences in trophic-level, depending on whether the contrasting δ^15^N values in the feathers reflect more than just the variation in baselines in the different areas.

In contrast, both South Georgia and common diving petrels spent the non-breeding period in waters either around the South Georgia archipelago or ~3000 km to the east-northeast. This pattern could be explained by their lower flight capability (higher wing loading) in comparison to blue petrels and Antarctic prions, restricting the capacity to travel far from the breeding colony^2^. The question remains as to how these two species avoid competing in the area of overlap. The answer seems to be that there are differences in environmental niche, and in trophic level; δ^15^N is higher in feathers of South Georgia than common diving petrel, which could reflect partial segregation by depth^2^,^14^, since these species prey mostly on copepods, which show an enrichment in ^15^N with depth^15^. Although the number of common and South Georgia diving petrels tracked was low, the consistency in spatial patterns among individuals of the same species, as well as the high consistency in feather stable isotope ratios between tracked birds and other sampled individuals, suggest that the spatial distributions were likely to be representative of the respective non-breeding population (see Figures S2 and S3 in the electronic supplementary material). However, this should be confirmed by further tracking.

In summary, our study demonstrates the advantages of integrating results from miniaturized devices and stable isotope analyses to compare spatial overlap and habitat use. It provides key insights into the ecological segregation of these highly abundant predators, particularly into the mechanisms that reduce competition for resources during the non-breeding period. Further investigations involving larger numbers of tracked individuals (particularly diving petrels) in future years would be useful for examining longer-term consistency in spatial, habitat and trophic overlap, and also for determining the potential repercussions of a reduction in abundance of Antarctic krill^16^, which is a key prey resource for many species during the non-breeding period.

## Material and Methods

### Fieldwork, geolocators and sampling

All work and methodologies were conducted in accordance to the approved guidelines of the Ethics Committee of BAS and Government of South Georgia and the South Sandwich Islands. Fieldwork was carried out at Bird Island, South Georgia (Fig. 1; 54°00’S, 38°03’W). To investigate non-breeding movements, miniaturized leg-mounted geolocators (MK18-model, 1.5 g; British Antarctic Survey, Cambridge, UK) were attached to 25 breeding adults of each species. Birds were captured by hand at marked nests during incubation in summer 2010/11, and devices retrieved in the following season (2011/12). Handling times were <3 min., and birds were always returned to burrows. Eight loggers were recovered from Antarctic prions, 11 from blue petrels, 3 from common diving petrels and 3 from South Georgian diving petrels. The non-breeding season was defined according to colony departure and return dates of tracked birds: Apr-Oct for Antarctic prions and South Georgia diving petrels; Feb-Sep for blue petrels and common diving petrels. Geolocation provides two positions per day, with a mean error ± SD of approximately 186 ± 114 km^17^. Geolocation data were analyzed and filtered using the BASTrak software suite following standard procedures^17^.

We collected 5–6 feathers from different parts of the mantle region from 13 blue petrels (11 tracked individuals), 11 Antarctic prions (8 tracked individuals), 12 South Georgia diving petrels (3 tracked individuals) and 10 common diving petrels (3 tracked individuals) to analyse δ^15^N and δ^13^C. Similar to other Antarctic and subantarctic petrels, these species moult body feathers during the non-breeding season, and so the isotopic composition (fixed at the time of synthesis) reflects diet and geographic range during that period^8^,^12^,^13^. Before isotopic determination at the Estación Biológica de Doñana^2^, feathers were cleaned using a 2:1 chloroform:ether rinse. To obtain integrated isotopic information for as much as possible of the non-breeding period^18^, all feathers from each individual were homogenised by powdering in a freezer mill prior to analysis.

### Environmental niche

The oceanographic variables used to quantify the habitat niche were two sea-surface temperature variables (SST; mean and fronts), chlorophyll-a (chl-a; mean and fronts), salinity, dissolved oxygen, depth, and distance to minimum sea-ice extent^11^. Variables were downloaded as raster grid layers from the Bio-ORACLE data set and then bilinearly resampled to the same resolution as the GLS positions (200 km; approx. 1.8°) using ESRI ArcGIS 9.3.1. These variables represent relevant annual metrics derived from monthly composite measures that have been proven useful for characterizing meso-scale habitat use^11^,^19^.

### Spatial, environmental and isotopic niche overlap

We calculated spatial, environmental (habitat) and isotopic niche overlap between species using the framework proposed by^20^, which applies kernel smoothers to species occurrence in a two-dimensional gridded space. This space was defined for the respective overlap analysis by the 200-km resolution Lambert equal-area X and Y coordinates of the entire non-breeding range (the 95% kernel density polygon derived from all GLS locations), by the first two axes of a principal component analyses (PCA) calibrated using the habitat variables over the whole non-breeding range, and by δ^15^N and δ^13^C values of all sampled feathers. Overlap was calculated using the *D-metric* which ranges from 0 (no overlap) to 1 (complete overlap). We applied a permutation-based approach (100 permutations) to evaluate whether the overlap values were higher than expected at random according to the available spatial, environmental and isotopic spac, respectively (similarity test^21^). For habitat analyses, the environmental availability for each species was constrained to that present in each species home-range (95% kernel density polygon). All analyses were conducted using the ‘ecospat’ library in R software. Mean δ^15^N and δ^13^C values were compared using ANOVA and Tukey posthoc tests.

## Additional Information

**How to cite this article**: Navarro, J. *et al.* Spatial distribution and ecological niches of non-breeding planktivorous petrels. *Sci. Rep.* **5**, 12164; doi: 10.1038/srep12164 (2015).

## Supplementary Material

Supplementary Information

## Figures and Tables

**Table 1:** Results comparing the overlap based on metric *D*, which ranges from 0 (no overlap) to 1 (complete overlap), in geographic space, environmental niche (PCA analysis) and isotopic space among blue petrels (BP), Antarctic prions (AP), South Georgia diving petrels (SGDP) and common diving petrels (CDP) from South Georgia during the non-breeding period.

Species	Spatial overlap	Niche overlap	Isotopic overlap
BP × AP	0.15	0.43*	0
BP × CDP	0.16	0.52*	0.15
BP × SGDP	0.10	0.24*	0
AP × CDP	0.08	0.49*	0.12
AP × SGDP	0	0.29*	0.06
SGDP × CDP	0.42*	0.34	0.14

^*^Similarity test p < 0.05.
