# Supplementary material for: Spatial distribution and ecological niches of non-breeding planktivorous petrels
Source: Sci Rep. 2015 Jul 13;5:12164. doi: 10.1038/srep12164 (PMC4499811; doi:10.1038/srep12164)
Supplement: Supplementary Information [file srep12164-s1.doc]

**ELECTRONIC SUPPLEMENTARY INFORMATION**

**Spatial distribution and ecological niches of non-breeding planktivorous petrels**

Joan Navarro^1,*^, Laura Cardador^1^, Ruth Brown^2^ and Richard A. Phillips^2^

^1^ Department of Conservation Biology, Estación Biológica de Doñana CSIC, Sevilla 41092, Spain

^2^ British Antarctic Survey, Natural Environment Research Council, Cambridge CB3 0ET, UK

*Author for correspondence:

Joan Navarro

e-mail: joan@ebd.csic.es

**Figure S1 and Table S1: Contribution of the oceanographic variables on the two principal axes of the PCA**


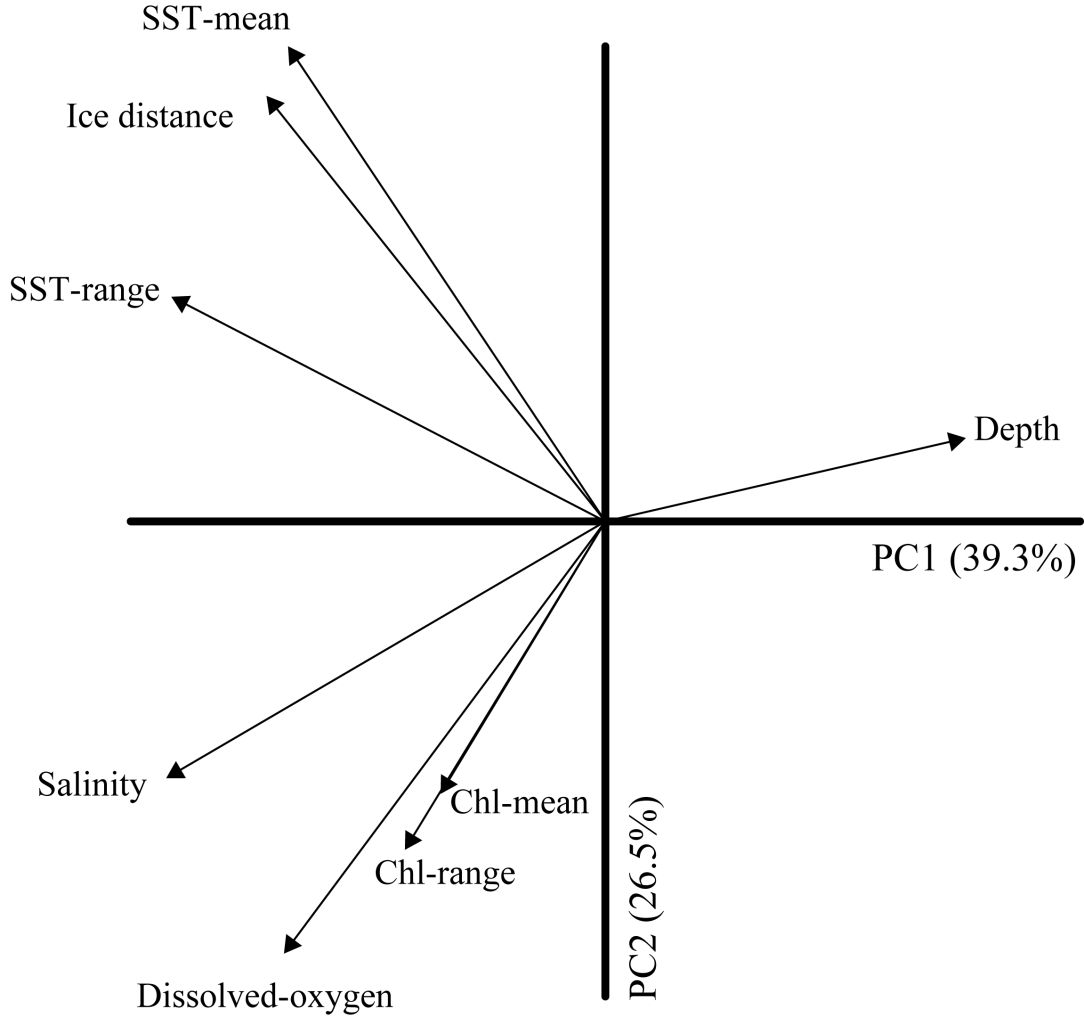


**Figure S1.** The contribution of the oceanographic variables on the two axes of the PCA and the percentage of variance explained by the two major axes (PC1 and PC2).

| **Table S1.** Factor loadings, proportion of variance explained by each oceanographic variable in PC1 and PC2 and correlation coefficient (Pearson’s Correlation) between PC1/PC2 and each oceanographic variable. PC1 and PC2 was derived from Principal Component Analysis (PCA) of habitats used during the non-breeding period by blue petrels, Antarctic prions, South Georgia diving petrels and common diving petrels tracked from South Georgia using geolocators in winter 2011. *P-values<0.05. | | | | |
| --- | --- | --- | --- | --- |
| Variable | Factor Loadings | | Correlation | |
|  | PC1 | PC2 | PC1 | PC2 |
| Depth | 0.381 |  | 0.01 | 0.69* |
| Salinity | -0.464 | -0.271 | 0.45* | -0.67* |
| Chlorophyll-*a* (Mean) | -0.212 | -0.346 | 0.15* | 0.89* |
| Chlorophyll-*a* (range) | -0.174 | -0.287 | 0.13* | 0.89* |
| SST (mean) | -0.335 | 0.501 | 0.98* | -0.01* |
| SST (range) | -0.458 | 0.237 | 0.75* | 0.43* |
| Dissolved-oxygen | -0.339 | -0.456 | -0.95* | 0.06* |
| Ice distance | -0.358 | 0.449 | 0.83* | -0.12* |
| Variance (%) | 39.22 | 26.46 |  |  |
| Cumulative Variance | 39.22 | 65.81 |  |  |

**Figure S2: Effect of the number of tracked diving petrel individuals on the spatial pattern observed**

We tested the relationship between the cumulative number of tracked individuals of each diving petrel species and various spatial metrics. In particular, we calculated the degree of spatial overlap (based on the *D-metric*; Warren 2008; Broennimann 2012) between paired data from common diving petrel-South Georgia diving petrel, common diving petrel-Antarctic prion, common diving petrel-blue petrel, South Georgia diving petrel-Antarctic prion and South Georgia diving petrel-blue petrel, including 1, 2 or 3 individuals. The results showed conclusively that spatial overlap was high between both diving petrel species, and was low between both diving petrel species, and Antarctic prion or blue petrel, independently of the number of individuals (Figure S2).


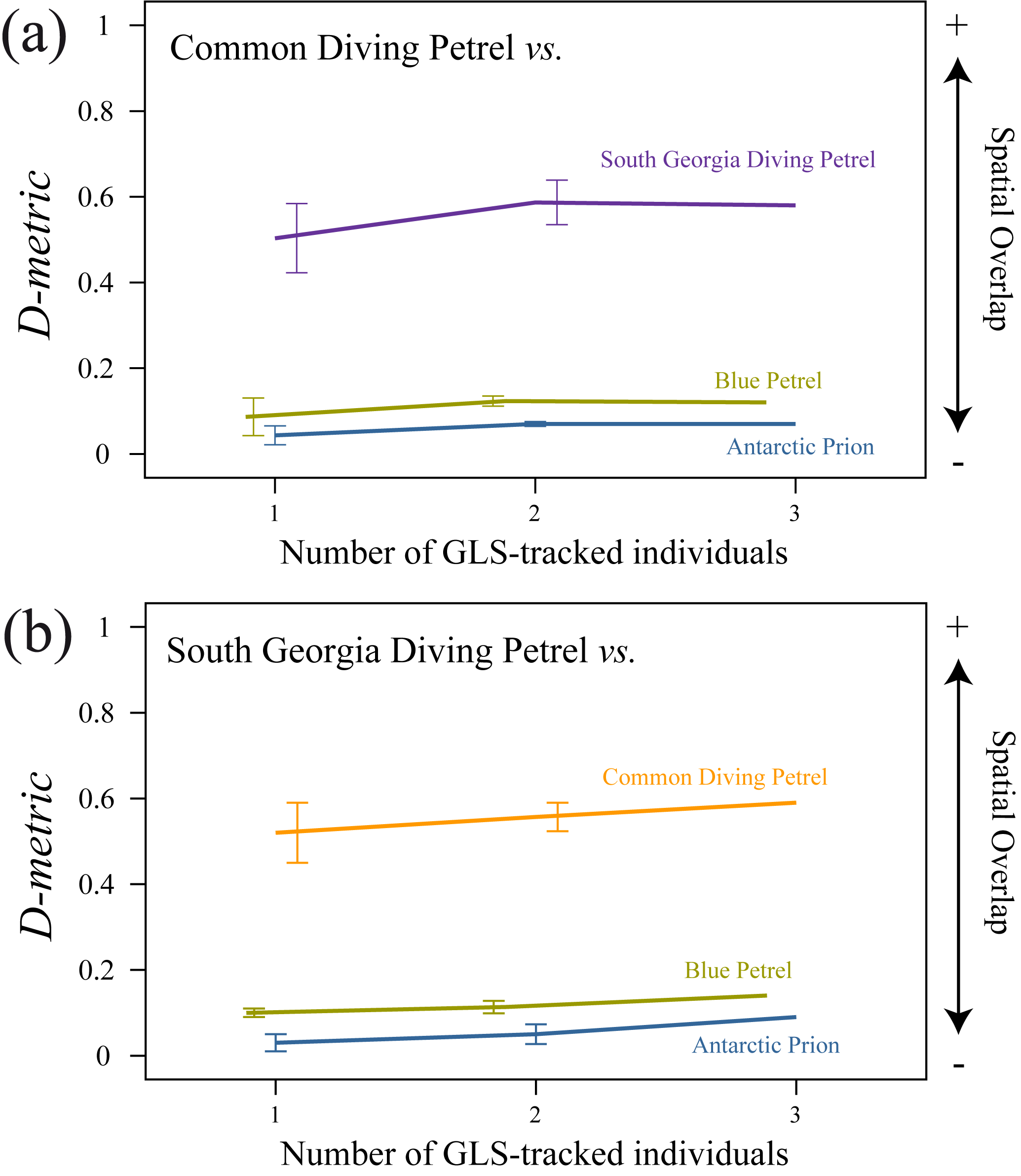


**Figure S2.** Cumulative spatial overlap (based on *D-metric*) between (a) common diving petrels and South Georgia diving petrels, Antarctic prions and blue petrels, and (b) South Georgia diving petrels and common diving petrels, Antarctic prions and blue petrels, in relation to the cumulative number of individual common or South Georgia diving petrels tracked using geolocators in winter 2011.

**Figure S3: Isotopic values from feathers collected during two consecutive years**

We analysed feather isotope data from 10 breeding individuals of each species sampled in the colony during the deployment year. These data are stable isotope ratios (reflecting carbon source - distribution - and trophic level) representing the previous non-breeding period (winter 2010). Isotopic niches based on these data are broadly similar in terms of overlap/segregation to the pattern observed during the GLS-recover year (Figure S3)


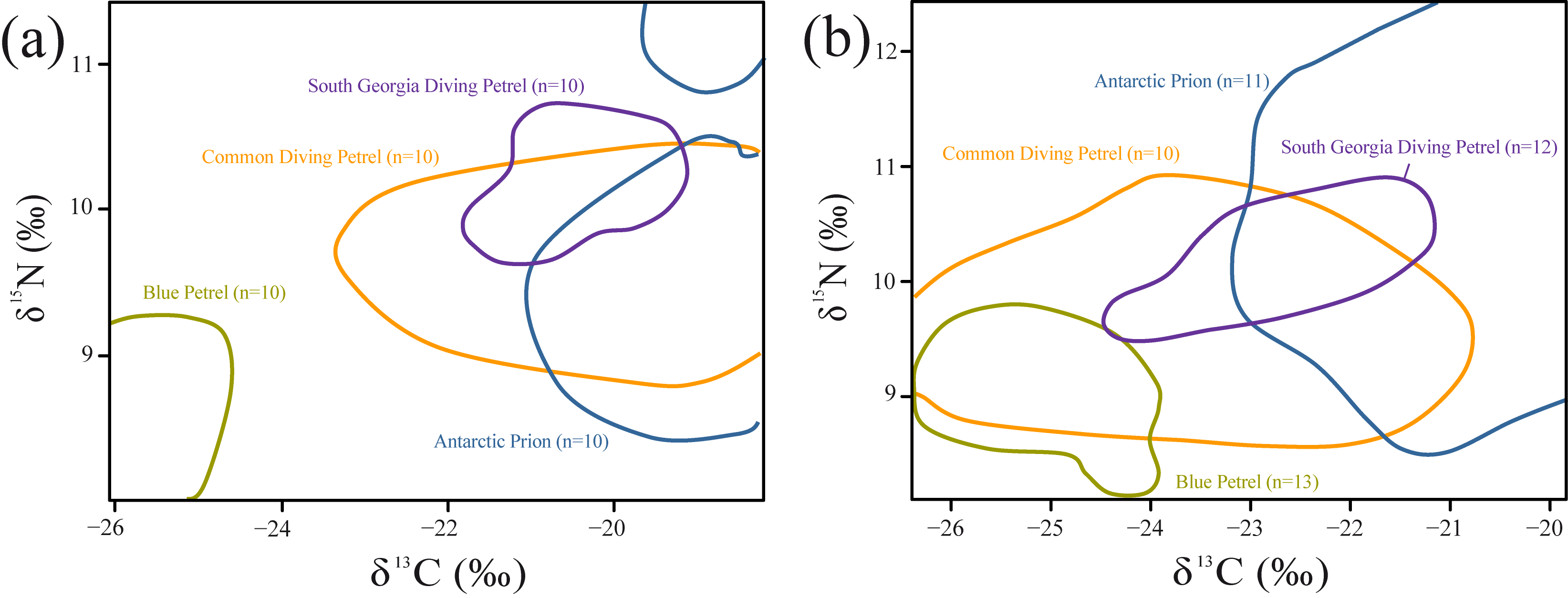


**Figure S3.** Isotopic niches (95% kernel density plots of δ^15^N and δ^13^C values in mantle feathers) of blue petrels, Antarctic prions, South Georgia diving petrels and common diving petrels from South Georgia during (a) non-breeding period in 2010, and (b) non-breeding period in 2011. Number of individuals for each species is also indicated.
